# Supplementary material for: Multiple Red Flags of Cardiac Amyloidosis in a Single Patient: Clinical Manifestations of an Underdiagnosed Disease
Source: Diagnostics (Basel). 2024 Dec 13;14(24):2812. doi: 10.3390/diagnostics14242812 (PMC11674418; doi:10.3390/diagnostics14242812)
Supplement: Supplementary file 1 [file diagnostics-14-02812-s001.zip › diagnostics-3362943-supplementary.pdf]

Table S1. 'Red flag' findings in amyloidosis. Based on the Position Statement of the European Society of Cardiology [1] and Polish Cardiac Society [2] experts with authors' modifications. Findings presented in the current paper are bolded.

<table>
<tr><th colspan="5">Cardiac Symptoms</th></tr>
<tr><th>Clinical Symptoms</th><th>ECG</th><th>ECHO</th><th>CMR</th><th>Biomarkers</th></tr>
<tr><td rowspan="3">Syncope, orthostatic hypotension</td><td rowspan="5">Atrioventricular conduction disease</td><td>Thickening of the LV and RV</td><td>Subendocardial LGE</td><td rowspan="5">Persistently elevated hs-cTn concentrations</td></tr>
<tr><td>LV diastolic dysfunction</td><td>Elevated T1 and T2 relaxation times</td></tr>
<tr><td>Enlargement of the RA</td><td rowspan="2">Challenges in selecting the inversion time</td></tr>
<tr><td>Symptoms of HF</td><td>Increased valve thickness</td></tr>
<tr><td>Family history of HF, PM, ICD</td><td>Thickening of the IAS</td><td>Increased extracellular volume</td></tr>
</table>

Progressive fatigue		Increased echogenicity		
**Resolution of hypertension, intolerance to previously tolerated antihypertensive medications**		**Aortic valve stenosis**		
	Pseudoinfarct pattern	Reduced longitudinal strain with apical sparing pattern	Abnormal gadolinium kinetics	**Disproportionally elevated NT-proBNP to degree of HF**
Hypotension or normotensive if previously hypertensive	Low/decreased QRS voltage or absence of hypertrophy features in the ECG, despite cardiac hypertrophy in the ECHO	**Granular sparkling of myocardium**		
	**Atrial fibrillation**	Pericardial effusion		
		**Non-cardiac symptoms**		
**Musculoskeletal system**	**Polyneuropathy**	**Gastrointestinal system**	**Urinary and reproductive system**	**Other Clinical Symptoms**
Carpal tunnel syndrome (bilateral)	Burning, stinging pain in the hands and feet	Chronic diarrhea or constipation	Urinary retention or incontinence	Dysautonomia

				Skin bruising
				Skin discoloration
				Cutis laxa
Lumbar spinal canal stenosis	Muscle weakness	Early satiety during meals	Erectile dysfunction	Macroglossia
Bicep tendon rupture, Popeye sign			Proteinuria	Deafness
**Pain/post-status after knee or hip joint arthroplasty**	Family history of polyneuropathy	Unintentional weight loss	Renal insufficiency	Vitreous deposits
Trigger finger				Corneal lattice dystrophy

Abbreviations: CMR, cardiac magnetic resonance; ECG, electrocardiogram; HF, heart failure; hs-cTn, high-sensitivity cardiac troponins; IAS, interatrial septum; ICD, implantable cardioverter defibrillator; LGE, late gadolinium enhancement; LV, left ventricle; NT-proBNP, N-terminal pro-B-type natriuretic peptide; PM, pacemaker; RA, right atrium; RV, right ventricle.
